# Stakeholders’ Perception on National Heatwave Plans and Their Local Implementation in Belgium and The Netherlands

**DOI:** 10.3390/ijerph13111120

**Published:** 2016-11-10

**Authors:** Joris Adriaan Frank van Loenhout, Jose Manuel Rodriguez-Llanes, Debarati Guha-Sapir

**Affiliations:** Centre for Research on the Epidemiology of Disasters (CRED), Institute of Health and Society, Université catholique de Louvain, Brussels, Belgium; jose-manuel.rodriguez-llanes@jrc.ec.europa.eu (J.M.R.-L.); debarati.guha@uclouvain.be (D.G.-S.)

**Keywords:** heatwaves, health protection, perception, key informant interviews

## Abstract

National heatwave plans are aimed at reducing the avoidable human health consequences due to heatwaves, by providing warnings as well as improving communication between relevant stakeholders. The objective of this study was to assess the perceptions of key stakeholders within plans in Belgium and The Netherlands on their responsibilities, the partnerships, and the effectiveness of the local implementation in Brussels and Amsterdam. Key informant interviews were held with stakeholders that had an important role in development of the heatwave plan in these countries, or its implementation in Brussels or Amsterdam. Care organisations, including hospitals and elderly care organisations, had a lack of familiarity with the national heatwave plan in both cities, and prioritised heat the lowest. Some groups of individuals, specifically socially isolated individuals, are not sufficiently addressed by the current national heatwave plans and most local plans. Stakeholders reported that responsibilities were not clearly described and that the national plan does not describe tasks on a local level. We recommend to urgently increase awareness on the impact of heat on health among care organisations. More emphasis needs to be given to the variety of heat-risk groups. Stakeholders should be involved in the development of updates of the plans.

## 1. Introduction

The effects of heatwaves on health in Europe have been investigated in a number of studies. An increase in mortality and years of life lost in European cities during the nineties has been observed, even after adjusting for the harvesting effect [1]. A main risk group for mortality consists of elderly people [2]. Although there is little evidence that directly describes the impact of heat on socially isolated individuals, including homeless people, they are considered a risk group due to the lack of social control and a relatively high proportion of morbidities. It was estimated that, due to the very severe heatwave in 2003 in Western Europe, between 1400 and 2200 individuals died in The Netherlands [3], and more than 70,000 in Europe [4]. Apart from mortality, heatwaves have a considerable impact on morbidity. Mastrangelo et al. reported an increase in respiratory diseases and heat diseases during heatwaves, but no increase in circulatory diseases [5]. Amongst the elderly, an increase in heat-related symptoms, such as fatigue, sleep disturbance and annoyance due to heat, was shown in a recent study by van Loenhout et al. as a consequence of increasing indoor temperatures [6]. Due to climate change, there is an expected increase in heatwaves in Western Europe, both in frequency and intensity [7].

The severe heatwave that hit Europe in 2003 prompted many countries to implement heatwave early warning systems with response plans [8]. These heatwave plans usually feature timely accurate warnings, tailored communications and notifications of adaptation actions to the most vulnerable populations and heat avoidance advice to general populations [9]. The main aim of the heatwave plans is to reduce the avoidable public health consequences of heatwaves. A study in the Florentine area (Italy) showed a general reduction in heat-related mortality from the four years before 2003 to the four years after 2003 in elderly (≥75 years) people [10]. In addition, a French study showed that mortality during a 2006 heatwave was lower than predicted by a model, which could partially be due to the introduction of a heat warning system [11]. A systematic review from 2014 looked at studies that assessed the impact of heat prevention plans and climate adaptation strategies, and found a reduction of adverse effects during extreme heat in places where preventive measures have been implemented [12]. In contrast, a recent study on heat-related mortality in nine European cities before and after the 2003 heatwave showed that improvement in adaptation was achieved by only a third of these cities, and two of these worsened their adaptation capacity while the other one remained unchanged [13].

Both Belgium and The Netherlands have developed national plans in 2005 and 2007, respectively, as public health measures against heatwaves [6,14]. In the Belgian region of Wallonia, a recent survey assessed the familiarity of stakeholders and end users with protection and adaptation measures to heat. However, no specific assessment of local implementation was undertaken [15]. Since the successful implementation of a national heatwave plan locally depends largely on the participation and collaboration of relevant stakeholders, we interviewed key informants from those organisations with the aim of assessing their perceptions on the heatwave plans in terms of responsibilities, partnerships, and effectiveness of the local implementation in Brussels and Amsterdam.

## 2. Materials and Methods

### 2.1. Desk Evaluation of National Heatwave Plans

A desk evaluation was performed on the national heatwave plans in Belgium and The Netherlands in December 2014. We evaluated whether the plans have described (i) main collaborating partners; (ii) different levels of alert; and (iii) stakeholders and responsibilities. For the topics that were included in the plans, we described the level of detail.

### 2.2. Key Informant Interviews

We decided to focus on stakeholders who had important roles in the development of the national heatwave plan in Belgium or The Netherlands, or its implementation in Brussels or Amsterdam. These cities were selected as major metropoles of Belgium and The Netherlands, respectively, where the effects of heatwaves are stronger due to the urban heat island effect [16]. In both countries, we contacted representatives at the following stakeholder organisations: national institutes of public health, regional health and environment agencies, municipalities, the Red Cross, elderly and homecare organisations, hospitals, overarching childcare centre networks, and circles of general practitioners. Key informants were identified through networks of the researchers, snowballing and internet searches. The aim was to interview stakeholders from each of three categories in each country, representing the hierarchical and communication organisation, which we defined as: (i) Activators, or those stakeholders who were (co-)responsible for developing and setting up the national heatwave plan in their country; (ii) Intermediaries, as those responsible for rolling out information from the activators to a (large) group of care providers and end users; and (iii) Care providers, those organisations or professionals directly responsible for health of risk groups with respect to heat. Stakeholders were contacted by email with a short explanation about the study, and the request to participate in an interview. Non-responders received a single reminder after 2–3 weeks, also by email.

An interview format was designed, containing the topics and questions for the key informant interviews (Appendix A). Interviews in The Netherlands were held in Dutch, and they were carried out between December 2013 and February 2014. In Belgium, interviews were conducted in Dutch or French, depending on the stakeholder’s language proficiency or preference, between March 2015 and May 2016. Interviews were not recorded to create a more informal interview situation. Instead, written notes were taken during the interview, and a written report was sent to the stakeholders afterwards for approval. For stakeholders who felt little affinity with the topic of heat and health and who did not see the need to participate in an interview, but still wanted to provide some input, a shortened version of the interview was administered via email. Thematic analysis was used to examine the data obtained in this study [17].

## 3. Results

### 3.1. Desk Evaluation of National Heatwave Plans

National heatwave plans for Belgium and The Netherlands were found on websites of the respective national governments [14,18].

The Belgian National Heatwave Plan is developed by the Federal Public Service for Health, Food Chain Safety and Environment (FOD). It is available in French as well as Dutch. Main collaborating partners that are described in the plan are the National Meteorological Institute (KMI), the Belgian Interregional Environment Agency (IRCELINE) and the Belgian regional governments (Flanders, Wallonia and Brussels). The heatwave plan is aimed at heatwaves as well as ozone, although the focus of our study was heatwaves. The plan contains information on heat-related health effects and their treatment, risk groups and aggravating factors. Risk groups for heat that are mentioned in the plan are young children, elderly, socially isolated individuals and persons who perform heavy physical exercise. In addition, different upscaling phases are described, as well as actions that will be taken during those phases (Table 1).

The Dutch National Heatwave Plan is developed by the National Institute for Public Health and the Environment (RIVM), in collaboration with the Ministry of Health, Welfare and Sport (VWS), the Royal Dutch Meteorological Institute (KNMI), the overarching organisation for Regional Public Health Institutes (GGD The Netherlands), the overarching organisation for healthcare institutes (ActiZ) and the Dutch Red Cross (NRK). The plan is available in Dutch and contains information on risk groups and situations, heat-related health effects, recommendations to prevent heat stress and communication strategies towards vulnerable groups. Risk groups for heat that are mentioned in the plan are elderly people living in care organisations, the chronically ill, socially isolated individuals, overweight people and children. Also, it describes stakeholders and their role in the plan, as well as different upscaling phases (Table 1).

The Dutch National Heatwave Plan seems more comprehensive than the Belgian plan. Sections that appear only in the Dutch plan are recommendations to prevent heat stress and communication strategies. In addition, the intended tasks of all stakeholders, also specifically during each of the upscaling phases, are described in the Dutch plan, but not in the Belgian plan (Table 2). The levels of alert and the criteria for activation differ between the two countries, since the two national heatwave plans were created independently from each other.

### 3.2. Key Informant Interviews

In The Netherlands, we were able to plan interviews with two activators of the national heatwave plan: the National Institute for Public Health and the Environment (RIVM), and the Dutch Red Cross (DRC). Intermediary organisations in Amsterdam that collaborated were the Municipal Health Service Amsterdam (MHSA) and the Municipality of Amsterdam (MoA). We contacted the biggest organisation on elderly care in Amsterdam, Cordaan, and they agreed to an interview as well. The biggest tertiary hospital in Amsterdam, the Academic Medical Centre (AMC) answered some of our questions by email, but did not participate in an interview. The total number of positive responses was six (Table 3).

In Belgium, we were able to carry out interviews with several activators of the national heatwave plan, namely the FOD public health, food safety and environment (FOD), Public Service Wallonia (PSW), and Leefmilieu Brussels (LB). Intermediary stakeholders whom we interviewed were from Red Cross Brussels (RCB) and two municipalities within the Brussels Capital Region, namely Etterbeek (MoE) and Saint-Gilles (MoS-G). A big homecare organisation in Brussels, Familiehulp, did not want to participate in an interview but answered some of our questions by email. The total number of positive responses was seven (Table 4).

Overall, it is noteworthy that most non-responding organisations belonged to the category of care providers (e.g., general practitioners, childcare centres).

#### 3.2.1. Familiarity with the National Heatwave Plans

We found that the three care organisations that participated in our study were not familiar with the existence of the national heatwave plan, both in Brussels and Amsterdam. With the exception of those, all other organisations were aware of the plan, although not always of the exact content (Table 3 and Table 4).

#### 3.2.2. Involvement in National Heatwave Plan Development

From the data collected, we detected that the national heatwave plans were developed without involvement of local organisations, including government, health and social care (Table 3 and Table 4).

#### 3.2.3. Heat as a Public Health Priority

Perception on heat as a priority for public health was observed as varying largely across stakeholders (Table 3 and Table 4). Heatwaves were considered by some stakeholders to be a lower priority than other public health risks, such as air pollution (MoS-G) and infectious diseases (Cordaan); heatwaves have a seasonal pattern of presentation and a low probability of occurrence (FOD); and that sufficient measures to cope with heatwaves were already in place (RIVM). On the other hand, reasons for placing heat as a high priority were that measures against heat are cost-effective and require a low investment (DRC); the likelihood of more frequent and severe heatwaves will increase due to trends in climate change (MHSA) and urbanisation (MoA); a direct increase in mortality can be observed during heatwaves (MoE); and heatwaves are perceived as more important than other public health issues, such as ozone (LB, RCB). Overall, care organisations did not give high priority to heat as a public health issue.

#### 3.2.4. Involvement in Warning At-Risk Populations

One observation was that some of the organisations directly involved in caring for heat-risk groups (Cordaan, Familiehulp) reported few or no activities aiming at warning these groups on the risks of heat. We also observed that, although a detailed description on all risk groups is available within national heatwave plans, some municipalities do not focus on all of them.

#### 3.2.5. Success in Reaching the At-Risk Populations

Perceived reasons were varied. Some stakeholders found the plan reached the at-risk population adequately due to a high level of media attention (RIVM) and others concurred that televised information adequately reaches targeted groups (FOD, RCB). Moreover, some stakeholders received positive feedback from regional public health organisations (RIVM) and citizens (MoS-G). In contrast, others felt that there is limited awareness from informants and risk groups on the topic of heat (DRC, MoE), and that the list of stakeholders involved in the care of risk groups was incomplete (LB). Homeless people did not fall under the responsibility of the municipality and were therefore excluded in municipal actions (FOD). The effectiveness of warning at-risk populations was not evaluated, according to a stakeholder interview (MHSA).

#### 3.2.6. Responsibilities Described in National Heatwave Plan

Dutch stakeholders reported overall that responsibilities were not clearly described and that the plan is non-committal. However, an updated version of the plan would be implemented in 2015, which would put more emphasis on describing tasks of different stakeholders. Similar results were found for Belgium, where most stakeholders felt that the national plan does not describe tasks on a local level.

#### 3.2.7. Collaboration between Stakeholders

We observed a clear trend in our results, suggesting that stakeholders on a higher level (i.e., activators) had a much more positive perception towards the quality of collaborations compared to intermediaries, which in turn were more positive than care organisations, the more local level of stakeholders considered in our study. This pattern was consistent across both study countries. One stakeholder pointed out that the quality of collaborations may suffer from the lack of sufficient budgets (MoE).

#### 3.2.8. Strengths and Weaknesses of the National Heatwave Plan

**Figure 1:**
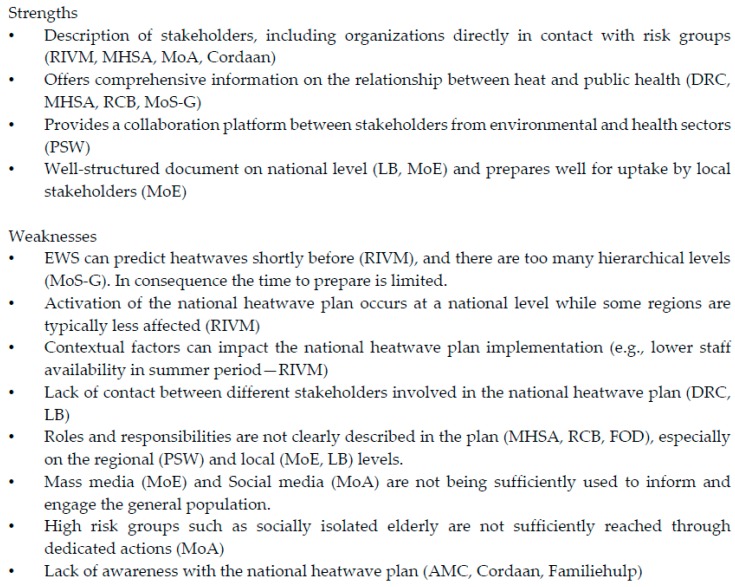
Observed strengths and weaknesses of the Belgian and Dutch National Heatwave Plans.

One strength consistently reported about the Dutch National Heatwave Plan was the inclusion of a description on relevant stakeholders. Both national heatwave plans were considered to offer a good evidence base on the links between heat and health. Especially for Belgium, stakeholders felt that the description of roles and responsibilities in the plan was not optimal. All care organisations reported lack of awareness with the plan as an issue (Figure 1).

## 4. Discussion

The most striking finding was the mismatch between the intended and the actual familiarity with the national heatwave plans among the care organisations under study. Even though elderly care institutes, homecare organisations and hospitals were listed in the national heatwave plans of both countries, representatives from these organisations were not aware of the existence of the plan, and did not receive alerts during a hot period. Additionally, among all respondents, care organisations gave the lowest priority to heatwaves. Both findings are consistent with a UK study, where a large majority of care organisations in London were not familiar with the national heatwave plan, even though these organisations were specifically mentioned in the Heatwave Plan for England [19], and the majority of these respondents did not regard heatwaves as high priority [20]. A study among care institutions in Amsterdam, which showed that less than 10% of the residents’ rooms in these institutions had air conditioning, suggests that heat is not considered an important factor for the health of this vulnerable population [21]. As care organisations have the closest contact with at-risk populations out of all the stakeholders, this low priority brings about a dilemma. Based on our findings, awareness of the impact of heat on health among stakeholders working in these types of organisations should be urgently addressed.

It became apparent that several risk groups for heat are not sufficiently addressed by the national heatwave plans. Homeless people in The Netherlands fall under the responsibility of the municipality, but in Belgium there is no governmental organisation responsible for them. However, homeless people are a risk group due to them having poorly controlled chronic diseases, respiratory diseases and mental illnesses, which render them vulnerable [22]. One stakeholder pointed out that individuals with little social contact do not receive enough attention in the national heatwave plan, and a survey held in the Wallonia region in Belgium came to a similar conclusion [15]. There is a discrepancy between the risk groups being targeted among the two municipalities in Belgium in our study: one targets all risk groups mentioned in the national heatwave plan, while the other focuses only on the elderly. We believe that there should be more emphasis on the variety of risk groups for heat, such as socially isolated individuals, and the organisational structures responsible for their care.

Although most stakeholders welcome the national heatwave plan, since it describes the different stakeholders and provides information on heat and health, it is considered a general weakness that the roles and responsibilities are not clearly described. Stakeholders can decide not to undertake any actions, since none of the intended actions is enforced by law. Although it is a conscious decision by the activators not to assign responsibilities, there is no consensus among the stakeholders that this is the best approach. The lack of contact between different stakeholders was also mentioned as a weakness by key informants from each country. We recommend the involvement of representatives from relevant stakeholders for a more effective uptake, as recent research suggests [15].

In Belgium, implementation on a local level is not included in the national plan, and should be taken up fully by the local stakeholders. Similarly, a previous study showed that the UK National Heatwave Plan, although considered an important source of disaster risk knowledge, was not successful in steering sustainable change in the way that heat risk is planned for at the local level [23]. This results in large variation in the number of activities between different municipalities, as was observed in our study. Sharing best practices and lessons learnt about implementation at a local level could be useful. For example, the municipality of Etterbeek had developed a very comprehensive local heatwave plan, in which they raised awareness towards risk groups, established a contact point for the general public and provided information to professionals. Lessons can be learnt from these pioneering municipalities by others. There is also a need for more detailed studies, describing the effectiveness of local heatwave plans in averting local excess mortality. Recent research has found no real adaptation to heat between the 2003 and 2007 heatwaves [13]. As this study shows, there is a substantial room for improvement in terms of local implementation of national heatwave plans, since these plans have a potential to improve adaptation to heat and heatwaves. The question then remains how well both countries are prepared to react to the next heatwave. As evaluation remains one of the largest gaps in research [12], this question is difficult to answer. However, this study does provide early insights into professional organisations that seem to be unaware of heat and its health impact, even though they tended to be those closest to the most vulnerable. This study uncovers the reality that information does not flow downstream and this might be an extraordinary source to avert morbidity and mortality in the future. To achieve this, we encourage local studies to be undertaken, which should include surveillance and evaluation.

## 5. Limitations

Our study does not give a complete overview on national heatwave plan perception in Brussels and Amsterdam, since only one or few key informants were interviewed for each type of organisation. Therefore, the results should be considered as indicative of the general situation. We did not get any insight into the extent of general practitioners and childcare centres that receive a heat warning or act after receiving it. The fact that we never received a reply from these organisations from either country could imply that they do not see this topic as a priority or simply have little knowledge about it.

Due to differences between our two studied countries, the responsibilities of parallel organisations might differ, e.g., the local/regional implementation of the national heatwave plan in Belgium is coordinated by the municipality, while in The Netherlands this falls under the responsibility of the Municipal Health Organisation. Therefore, a valid comparison of national heatwave plan perception between the two countries is not always possible. However, we would still encourage Belgium and The Netherlands to collaborate, e.g., by streamlining the phases and activation criteria for the national heatwave plans.

## 6. Conclusions

**Figure 2:**
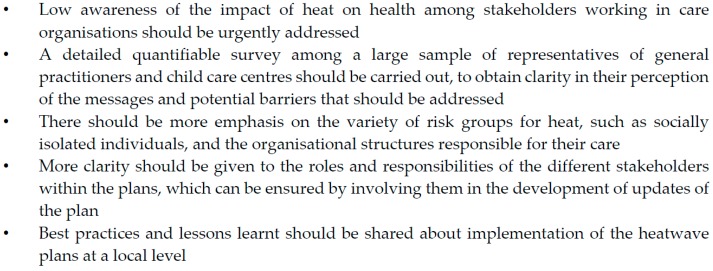
Recommendations related to national heatwave plans based on this study.

We found a lack of familiarity with the national heatwave plan among care organisations in both Belgium and The Netherlands. Among all stakeholders, care organisations also prioritised heat the lowest. We recommend urgently increasing awareness on the impact of heat on health among these stakeholders, which should be the priority of the activators in both countries. Some groups of individuals, such as the homeless and other socially isolated individuals, do not seem to be sufficiently addressed by the current national heatwave plans and most local plans, which is why more emphasis needs to be given to the variety of heat-risk groups and how to reach them. More clarity should be given to the roles and responsibilities of the different stakeholders within the plans. This can be ensured by involving them in the development of updates of the plan. Implementation of the plan on a local level is not described in the Belgian National Heatwave Plan, and its success depends fully on the priority that is given to it by local stakeholders. Sharing best practices could be a way to achieve more harmonised and higher quality plans, which are fully applicable in real settings, taking into consideration that resources vary between municipalities. Finally, due to several representatives being excluded from our study, there is a lack of insight in how messages from the national heatwave plans are perceived by e.g., general practitioners and childcare centres. We suggest a detailed quantifiable survey among a large sample of representatives from these groups, to obtain clarity in their perception of the messages and potential barriers that should be addressed by activators of the national heatwave plans. The main recommendations from this study are summarized in Figure 2.

## Figures and Tables

**Table 1:** Overview of the different levels of alert in the Belgian and Dutch National Heatwave Plans.

	Belgium			The Netherlands		
Levels of Alert	Phase	Activation	Actions for Main Stakeholders	Phase	Activation	Actions for Main Stakeholders
	Watchfulness	15 May until 30 September	- Informing the public - Spreading an information leaflet on heat	Watchfulness	1 June until 1 September	- Preparing for a hot period - Raising awareness among employees
	Warning level 1	- Minimum temperature over two days >18 °C - Maximum temperature over two days >30 °C	- Preparing warning and alert messages	Pre-warning	Odds of five days >27 °C higher than 20%	- Informing national organisations and regional information points (RPHSs) (i.e., National Institute for Public Health and the Environment (RIVM)) - Checking whether preparation for a warning phase are in order
	Warning level 2	- Minimum temperature over three days >18 °C - Maximum temperature over three days >30 °C	- Informing professionals, including general practitioners, hospitals, elderly care, homecare - Initiating a media campaign - Initiating a call centre	Warning	Odds of five days >27 °C higher than 90%	- Press release for general population (RIVM and Royal Dutch Meteorological Institute (KNMI)) - Sending warning message to intermediaries (RIVM) - Creating a regional information point (RPHSs)
	Alert	- Same criteria as in warning phase level 2 AND - Ozone thresholds are reached	- Intensifying previous measures - Creating a crisis centre			

**Table 2:** Overview of the different stakeholders included in the Belgian and Dutch National Heatwave Plans.

	Belgium		The Netherlands	
Stakeholders	Type	Tasks	Type	Tasks
	General practitioners	No tasks specified	Municipalities	- Proactively supporting vulnerable groups (e.g., homeless and drug users) - Opening a counter for providing information to the public
	Hospitals	No tasks specified	Regional Public Health Services (RPHSs)	- Providing information on a hot period to municipalities, elderly care, general practitioners and volunteer organisations - Creating an information point for the general public, professionals and volunteers
	Elderly care	No tasks specified	General practitioners	- Answering questions and providing information to vulnerable groups - Signaling heat-related symptoms in patients
	Homecare	No tasks specified	Pharmacies	- Providing advice to the public on dealing with heat - Providing information about risks of heat in combination with certain medication
			Elderly care	- Developing an internal heat plan, which includes measures to reduce harmful effects of heat on residents
			Homecare	- Signaling an increased demand in care
			Non-Governmental Organisations	- Offering additional support to vulnerable groups

**Table 3:** Overview of interviews with selected key informants in The Netherlands.

Country	Role in NHP	Organisation Name	Organisation Type	Familiarity with NHP	Role within NHP	Heat as a Public Health Priority	Involved in Warning the At-Risk Population	Successfulness in Reaching the Risk Population	Responsibilities Described in NHP	Collaboration between Stakeholders
The Netherlands	Activators	National Institute for Public Health and the Environment (RIVM)	National government	Yes	Plan development and activation, awareness through media, contact point for professionals	Medium	Yes (all risk groups)	Yes	No	Good
		Dutch Red Cross (DRC)	NGO	Yes	Input on plan development, awareness through media (i.e., press releases), mobilisation of volunteers	High	Yes (high risk groups)	Partially	No	Partial (limited involvement in national heatwave plan development)
	Intermediaries	Municipal Health Service Amsterdam (MHSA)	Regional government (public health)	Yes	Providing information and advice to professionals	High	Indirectly	Unknown	No	Partial (adequate communication with RIVM, more difficulties in communication with GPs)
		Municipality of Amsterdam (MoA)	Local government	Yes, but not with content	Care for certain vulnerable populations (homeless persons, drug users), contact point for general public	High	No	Not applicable	Not reported	Partial (collaboration with MHSA should be improved)
	Care organisation	Academic Medical Centre (AMC)	Hospital	No	Not reported	Low	No response	No response	No response	Poor (lack of collaboration with other stakeholders)
		Cordaan	Elderly care and homecare	No	Care for certain vulnerable populations (e.g., elderly, young children, handicapped)	Medium	Yes (elderly, only at care centres)	Yes	Unknown	Poor (lack of collaboration with other stakeholders)

NHP: National Heatwave Plan.

**Table 4:** Overview of interviews with selected key informants in Belgium.

Country	Role in NHP	Organisation Name	Organisation Type	Familiarity with NHP	Role within NHP	Heat as a Public Health Priority	Involved in Warning the At-Risk Population	Successfulness in Reaching the Risk Population	Responsibilities Described in NHP	Collaboration between Stakeholders
Belgium	Activators	Public Service Wallonia (PSW)	Regional government	Yes	Input on plan development, providing information to professionals	Medium (health sector), High (social sector)	Indirectly	Yes	Yes	Good (social sector), Poor (health sector)
		Leefmilieu Brussel (LB)	Regional government (environment and health)	Yes	Input on plan development, coordination of regional implementation	High	Indirectly	No	No	Good
		FOD Public health, food safety and environment (FOD)	National government	Yes, but not with content	Commissioned the plan, awareness through media, contact point for professionals, providing advice to professionals	Medium	Yes (all risk groups)	Yes	No	Good
	Intermediaries	Red Cross Brussels (RCB)	NGO	Yes, but not with content	Follow instructions from FOD, providing information to professionals	High	Indirectly	Yes	No	Partial (good collaboration on other issues than heat)
		Municipality of Etterbeek (MoE)	Local government	Yes	Coordination of local heatwave plan, awareness towards risk groups, contact point for general public, providing information to professionals	High	Yes (all risk groups)	Partially	No	Partial (lack of involvement of some stakeholders)
		Municipality of Saintt-Gilles (MoS-G)	Local government	Yes	Coordination of local heatwave plan	Medium	Yes (elderly)	Yes	No	Partial (low awareness of responsibilities of other stakeholders)
	Care organisation	Familiehulp	Homecare	No	Care for own employees	No response	No	Not applicable	No response	Poor (lack of collaboration with other stakeholders)

NHP: National Heatwave Plan.

## Interview Format for Stakeholders with a Role in National Heatwave Plans

### Current Roles and Obligations

1 Are you familiar with the content of the National Heat Plan?
2 Was your organisation involved in the development of the plan?
3 Are there other heat plans (e.g., local, organisational) that include your organisation?
4 What role/tasks does your organisation perform with respect to heat exposure, in addition to what is described within the National Heat Plan?
5 How high would you prioritise exposure to heat as a public health threat (e.g., on a scale of 1−10)?

### Message to the Public

1 Is your organisation directly involved in warning a population at risk for adverse effects due to heat? If yes:
  What are your target groups?
  Do you feel that you reach a large proportion of these groups?
  Do you feel the National Heat Plan offers enough options to adequately reach this population?
  Do you feel another way of reaching the target populations might be more effective?
2 Do you feel the messages and recommendations that the National Heat Plan presents are sufficiently clear for the population at risk and their caregivers?
3 How well do you think the population at risk changes its behaviour due to recommendations from the heat plan (e.g., on a scale of 1–10)?

### Cooperation with Other Stakeholders

1 Which other organisations are you in contact with on the topic of heat:
  During a cold/normal period?
  When the heat plan is activated?
2 Are all these collaborations described in one of the heat plans?
3 Do you feel the responsibilities of the different organisations are clearly described in the heat plans?
4 Do you feel the communication/cooperation between the different stakeholders functions well?
5 Do you feel that the current number of collaborations is sufficient?
  If yes: do you think the number of collaborations should be reduced to simplify the system?
  If no: which collaboration is currently not included in a heat plan, but would, in your opinion, be an important addition?

### Future Roles

1 Are you aware of the impact that climate change will have on your country, with respect to heat exposure?
2 Is there/has there been a discussion within your organisation on adaptations that might be needed to cope with the impact of climate change on the intensity and frequency of heat waves?
3 Do you think the current heat plan will suffice when the intensity and frequency of heatwaves will increase, in terms of collaborations with other organisations? If no: Which additional collaborations do you feel would be necessary?

### General Conclusion

1 What do you consider to be strong aspects of the national heat plan?
2 What do you consider to be weak aspects of the national heat plan?
3 Do you have any other remarks which could be relevant to our project?
